# Non-narcotic Perioperative Pain Management in Prosthetic Breast Reconstruction During an Opioid Crisis: A Systematic Review of Paravertebral Blocks

**DOI:** 10.1097/GOX.0000000000002299

**Published:** 2019-06-14

**Authors:** Hanae K. Tokita, Thais O. Polanco, Meghana G. Shamsunder, Stefan Dabic, Vaidehi G. Patel, Robert J Allen, Joseph H. Dayan, Babak J. Mehrara, Evan Matros, Jonas A. Nelson

**Affiliations:** From the Section of Plastic and Reconstructive Surgery, Memorial Sloan Kettering Cancer Center, New York, N.Y.

## Abstract

**Background:**

Alternatives to postoperative, narcotic pain management following implant-based, postmastectomy breast reconstruction (IBR) must be a focus for plastic surgeons and anesthesiologists, especially with the current opioid epidemic. Paravertebral blocks (PVBs) are a regional technique that has demonstrated efficacy in patients undergoing a variety of breast cancer–related surgeries. However, a specific understanding of PVB’s efficacy in pain management in patients who undergo IBR is lacking.

**Methods:**

A systematic search of PubMed, EMBASE, and Cochrane Library electronic database was conducted to examine PVB administration in mastectomy patients undergoing IBR. Data were abstracted regarding: authors, publication year, study design, patient demographics, tumor laterality, tumor stage, type, and timing of reconstruction. The primary outcome was PVB efficacy, represented as patient-reported pain scores. Secondary outcomes of interest include narcotic consumption, postoperative nausea and vomiting, antiemetic use, and length of stay.

**Results:**

The search resulted in 1,516 unique articles. After title and abstract screening, 29 articles met the inclusion criteria for full-text review. Only 7 studies were included. Of those, 2 studies were randomized control trials and 5 were retrospective cohort studies. Heterogeneity of included studies precluded a meta-analysis. Overall, PVB patients had improved pain control, and less opioid consumption.

**Conclusion:**

PVBs are a regional anesthesia technique which may aid in pain management in the breast reconstructive setting. Evidence suggests that PVBs aid in controlling acute postoperative pain, reduce opioid consumption, and improve patient length of stay. However, some conflicting findings demonstrate a need for continued research in this area of pain control.

## BACKGROUND

The number of implant-based postmastectomy reconstruction (PMR) procedures has steadily increased by 11% per year in the United States, being the most commonly utilized modality of PMR.^1^ Postmastectomy breast reconstruction is associated with an improved quality of life, with implant-based (IBR) or tissue expander (TE) reconstruction being a less invasive option, resulting in the shortest recovery time.^2–5^ Despite the cited advantages to prosthetic PMR, patients are at risk for significant postoperative acute and chronic pain.^6–9^ Because severe, acute postoperative pain is a major risk factor for patients to develop chronic pain, proper pain management techniques are necessary for maintaining positive quality of life following breast reconstruction.^10–12^

Due to the current opioid epidemic in the United States, the use of opioid analgesics has faced intense scrutiny.^13^ Between 2010 and 2015, the estimated opioid overdose death rate increased by 156%,^14^ where 21%–29% of patients misused opioids prescribed for pain management.^15^ Surgeons routinely prescribe opioids postoperatively and are responsible for an estimated 9.8% of all opioid prescriptions in the United States.^16^ Therefore, postoperative pain management may play a role in the opioid epidemic, especially for opioid-naive patients. Opioid prescriptions to opioid-naive patients following minor surgery are increasing,^17–21^ with these patients having a 4.1% chance of chronic opioid use at 1 year.^22–25^ Furthermore, among surgical patients, female gender remains a potential factor for chronic opioid usage due to exhibiting more pain.^3,26–30^

**Fig. 1.**
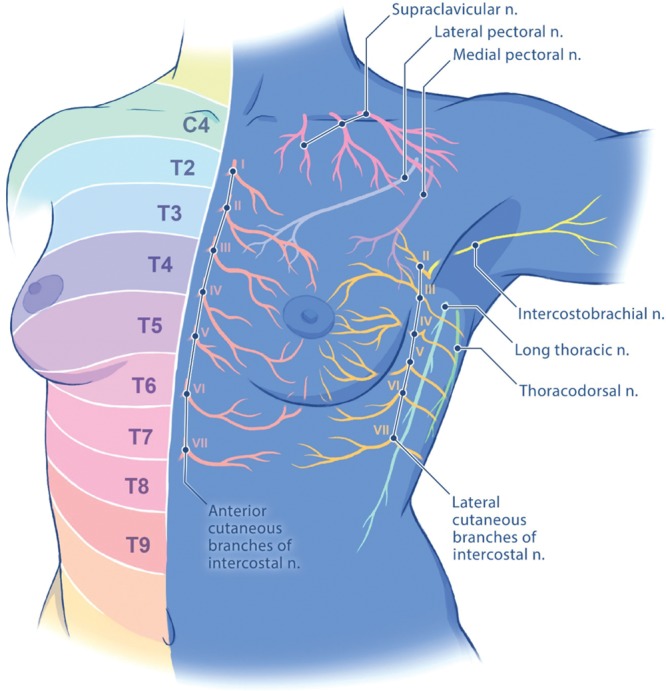
Chest wall innervation. n., nerve.

**Fig. 2.**
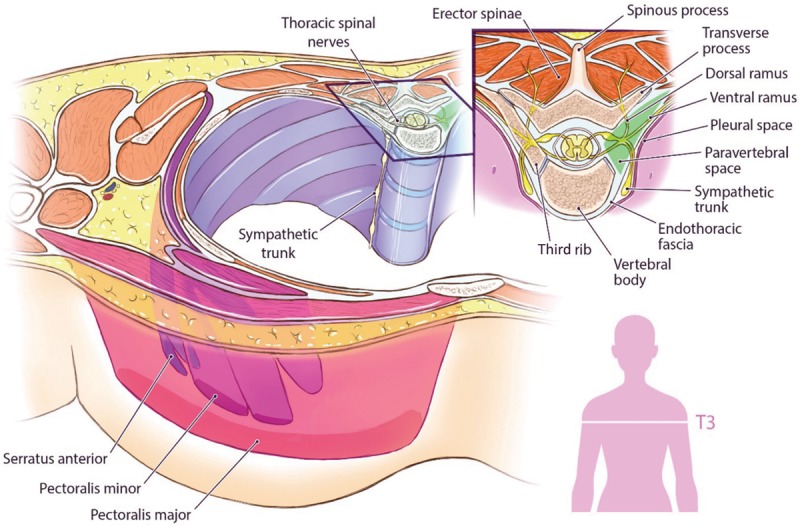
Anatomy of paravertebral space.

Such risk factors may contribute to opioid misuse in IBR patients with poorly controlled pain. To improve upon postoperative pain management following PMR in the context of a national, opioid epidemic, it is timely and necessary to understand alternative options. First described in the early 1900s, thoracic paravertebral blocks (PVBs) are a regional anesthetic block where local anesthesia is injected adjacent to thoracic vertebra and beneath the costotransverse ligament to block thoracic spinal nerves after they emerge from the intervertebral formina (Figs. 1, 2). PVBs have been shown to decrease postoperative pain and opioid consumption, limit postoperative nausea and vomiting (PONV), and reduce length of stay (LOS) in patients undergoing a variety of breast cancer surgeries.^31–33^ However, there is little evidence and consensus on the efficacy of PVBs in breast cancer patients undergoing prosthetic PMR. The purpose of this study was to conduct a systematic review to assess the effectiveness of PVB in IBR patients with a diagnosis of breast cancer.

## METHODS

### Data Sources and Search Strategy

This protocol has been registered with the National Institute of Health Research: Prospective Register of Systematic Reviews (PROSPERO; Registration No. CRD42018115156). We reported in accordance with the preferred reporting items for systematic reviews and meta-analysis guidelines.

A comprehensive search was performed using PubMed, EMBASE, and Cochrane Library electronic databases with publication end date of October 2018. No language restrictions were applied. The bibliographies of selected articles were manually checked for relevant references.

### Inclusion and Exclusion Criteria

Studies were included if they described PVB use in prosthetic PMR (TE or implant) following immediate or delayed mastectomy, among women older than 18 years with a diagnosis of breast cancer. Review articles, case reports, conference or meeting abstracts, editorial comments, studies describing PVB for mastectomy only, autologous tissue-based breast reconstruction (ABR), cosmetic breast surgery, and male patients were excluded.

### Abstraction

Two authors (T.P. and S.D.) independently assessed all titles and abstracts. Studies were subjected to full-text review by 2 authors (T.P. and S.D.) if at least 1 reviewer marked it for inclusion based on the above inclusion criteria. Consensus was reached when all reviewers agreed to include or exclude the study.

Data abstraction for the following were retrieved: authors, publication year, study design, patient demographics, tumor laterality, tumor stage, type and timing of reconstruction, and comorbidities. The primary outcome of interest for review was PVB efficacy at pain control. Secondary outcomes of interest included PVB-related complications, morphine consumption, PONV, antiemetic use, and LOS.

### Quality Assessment

Two authors (T.P. and S.D.) performed an independent assessment of the methodological quality and risk of bias in the included studies using the Cochrane Collaboration Risk of Bias tool (randomized trials)^34^ and the methodological index for nonrandomized studies (MINORS, observational studies).^35^

### Data Synthesis

Although all of the articles included an adequate endpoint relevant to our analysis in this review, there was significant variation in the format in which outcomes were reported. As a result, meta-analysis was not conducted and comparisons among studies were made in a qualitative manner, grouped by outcomes of interest to this study.

## RESULTS

**Fig. 3.**
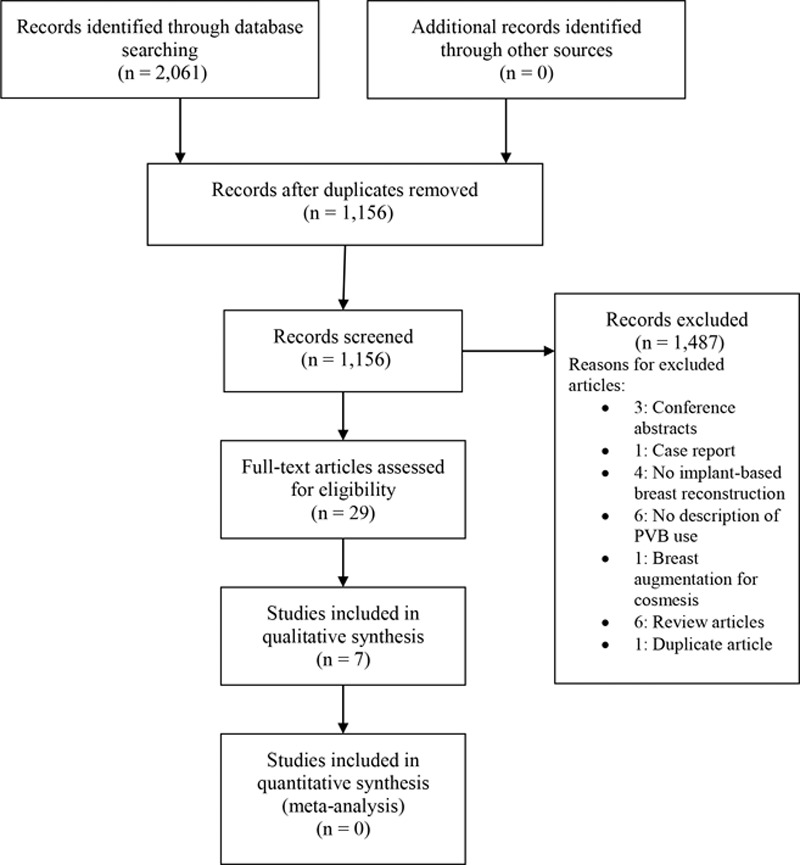
Preferred reporting items for systematic reviews and meta-analysis flow chart (PRISMA). PRISMA, preferred reporting items for systematic reviews and meta-analysis.

The systematic search resulted in 1,516 unique articles. After screening by title and abstract, 29 articles met the inclusion criteria for full-text review. A total of 7 studies were included. (Fig. 3) Two studies were randomized control trials (RCTs)^36,37^ and 5 were retrospective reviews.^33,38–41^ The combined, 7 studies resulted in a total of 877 patients, of which 478 had administration of PVB (Table 1). Two studies investigated PMR with TE only^33,36^ whereas 1 study focused only IBR.^39^ The remaining 4 studies included both TE and IBR; only one of these studies reported PVB use in direct TE-to-implant exchange.^42^ PVB effectiveness was compared with general anesthesia (GA) alone in 6 studies^33,36–39,41^ with the seventh study comparing PVB with liposomal bupivacaine (LB) wound infiltration.^40^ All 5 of the retrospective cohort studies used ultrasound-guided administration of PVB,^33,38–41^ whereas both RCTs used anatomical landmarks to administer PVB.^36,37^ Regarding the administration of PVB, 5 studies reported multilevel injections,^36–38,40,41^ whereas the remaining 2 studies reported single-level injections.^33,39^ Due to heterogeneity in outcome definitions, it is unclear if level of administration impacts postoperative pain or opioid use. All 5 of the retrospective cohorts used bupivacaine in the PVB procedures^33,38–41^; however, 1 RCT used levobupivacaine^37^ and the other RCT used ropivacaine.^36^

**Table 1.**
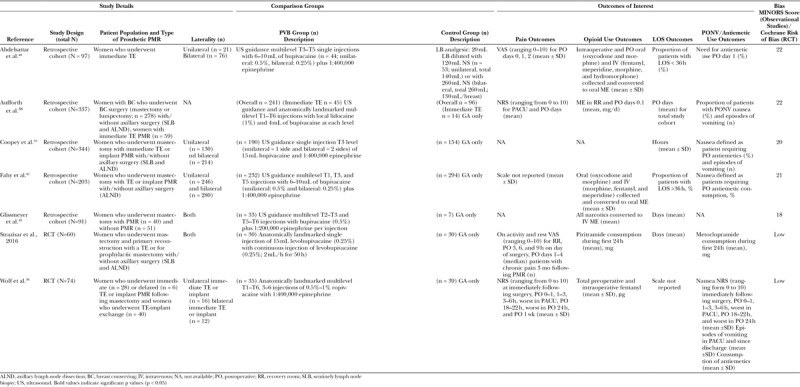
Study Characteristics

### Quality Assessment

Quality assessment of the cohort studies using modified MINORS score yielded a range of 18–22. The most common deficiency was lack of prospective calculation of sample size, likely due to the retrospective nature of these studies. The 2 RCTs were reviewed according to Cochrane’s Risk of Bias tool and deemed to have low bias.

### Postoperative Pain Outcomes

Pain scores were reported in 3 studies (Table 2). One study used average Numeric Rating Score (NRS; 0–10 numeric pain rating scale) and 2 studies used the visual analog scale (VAS; 0–10 numeric pain rating scale). Wolf et al. reported significantly lower average NRS scores among PVB patients compared with GA-alone patients for the postoperative intervals of 0–1, 1–3, and 3–6 hours.^36^

**Table 2.**
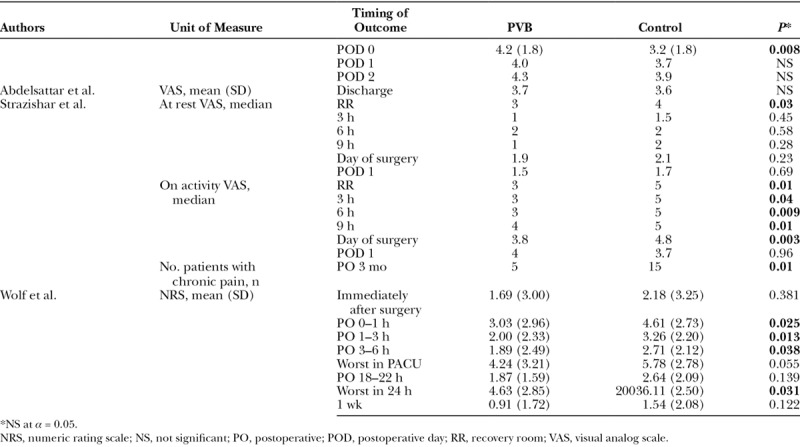
Postoperative Pain Measure Results

Strazisar et al. and Abdelsattar et al. evaluated pain using the VAS.^37,40^ Strazisar et al. studied changes in postoperative pain stratified by activity levels. Resting PVB patients had a significantly lower median VAS score compared with GA-alone patients only in the recovery room. However, “on activity” (activity with the upper extremity), PVB patients had significantly lower median pain scores than GA-alone patients at all postoperative time periods (from recovery room to 9 hours, at 3-hour intervals). On activity, median VAS scores on the day of surgery were significantly lower for PVB patients than for GA-alone patients; there was no significant difference in median scores for the first postoperative day between these groups.^37^ Abdelsattar et al. compared a PVB treatment group to a LB control group, reporting a significantly lower, average VAS pain score only at day 0 among the LB group versus the PVB group. The pain scores were not significantly different for the other postoperative assessment days.^40^

### Opioid Use Outcomes

Opioid use was reported as consumption of narcotics or morphine equivalents (MEs) in 6 of the 7 studies (Table 3). Both RCT studies showed that PVB patients received less postoperative opioids compared with GA-alone patients.^36,37^ Specifically, Strazisar et al. reported that on average, patients who received PVB with levobupivacaine consumed significantly less piritramide in the first 24 hours after surgery compared with GA-alone patients.^37^ Wolf et al. reported average, total fentanyl administered preoperatively and intraoperatively, noting that PVB patients were administered less fentanyl compared to patients with GA alone.^36^

**Table 3.**
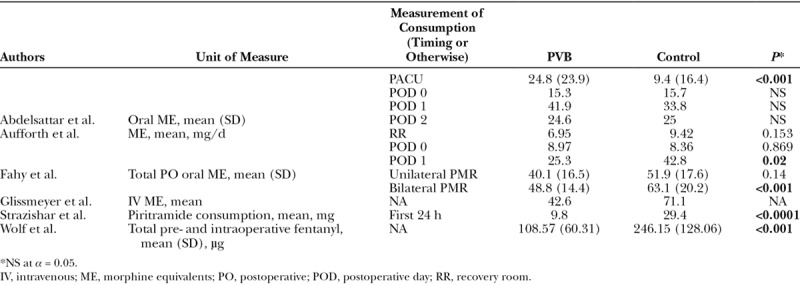
Consumption of Opioids

Additionally, 2 retrospective studies showed that PVB patients received less postoperative narcotics compared with the comparator. For patients undergoing bilateral PMR, Fahy et al. reported on average, PVB patients consumed significantly less ME than those given GA alone; however, this difference was not observed in patients undergoing unilateral mastectomy with PMR.^41^ Aufforth et al. also reported that PVB patients who underwent immediate TE PMR consumed less ME than GA-alone patients during the first postoperative day; however, this difference was not seen between the 2 groups in the recovery room or 0 days following surgery.^38^

Glissmeyer et al. reported that PVB patients received less ME compared to non-PVB patients; however, no statistical analysis was conducted.^39^ Lastly, Abdelsattar et al. found that PVB patients consumed a significantly higher average of ME compared to LB-only patients in the postanesthesia care unit (PACU) but had no significant difference in postoperative opioid consumption days 0–2.^40^

### Length Of Stay

Two studies reported LOS as proportion of patients with a LOS less than 36 hours. Abdelsattar et al. reported no significant difference in LOS between the LB group versus the PVB group.^40^ Similarly, Fahy et al. reported no significant difference in LOS between patients with PVB compared to GA alone/non-PVB patients, following a multivariate analysis.^41^

One study reported LOS in hours while 2 studies measured LOS in days. Coopey et al. reported that, on average, patients undergoing prosthetic PMR with PVB had a significantly shorter LOS compared to the non-PVB group, regardless of laterality (unilateral versus bilateral) or type (TE versus immediate implant placement) of PMR.^33^ Strazisar et al. reported that there was no significant difference in LOS between the local PVB group and the standard treatment group.^37^ Glissmeyer et al. reported PVB patients had shorter LOS (1.3 days) compared to those without PVB (2 days); however, this study did not perform a statistical analysis to determine significance.^39^

Lastly, Wolf et al. reported that on general LOS, no significant difference in length of hospital stay was found between the PVB treatment and control groups.^36^

### PONV and Antiemetic Medication

Wolf et al. investigated postoperative nausea scores (reported as NRS ranging from 0 to 10), reporting no significant difference in average scores between the PVB group and the GA-alone group at various intervals (immediately following surgery and up to 22 hours postoperatively). Additionally, they reported no significant difference in the average number of vomiting episodes in the PACU or at time of discharge between the 2 groups.^36^

Fahy et al. used postoperative antiemetic administration as a proxy for postoperative nausea. Analyses showed the PVB group receiving significantly less antiemetics than the GA-alone group, even after controlling for laterality of PMR.^41^ Coopey et al. reported a significantly lower proportion of patients with postoperative nausea in the PVB group compared to the non-PVB group, but found no significant difference in the proportion of patients with postoperative vomiting. However, this study did not quantify antiemetic use between the 2 groups.^33^

Abdeslsattar et al. reported a significantly lower proportion of LB patients using antiemetic medication one day following PMR compared to PVB patients.^40^ Strazisar et al. also reported that PVB patients on average consumed significantly less metoclopramide than GA patients.^37^ Lastly, Wolf et al. found no significant difference in average consumption of antiemetics among PVB patients compared to GA-alone patients.^36^

## DISCUSSION

In light of the US opioid epidemic, there is an intense focus on finding alternatives to effective postoperative pain control following surgery.^25,42–46^ In 2016, the CDC released clinical guidelines for chronic pain management, aiming to decrease misuse, abuse, and overdose from opioids. However, there remains minimal guidance for physicians on the management of acute pain, specifically in the perioperative and acute postoperative setting.^47,48^ Regional anesthetic techniques, such as PVBs, are an example of a possible method to reducing postoperative pain and initial narcotic utilization with well described, minimal complications.^49^ This review demonstrates that this technique not only may favor positive postoperative outcomes, but also displays the heterogeneity of existing studies and their slightly conflicting results.

PVB was shown to positively reduce acute postoperative pain and reduce PONV/antiemetic medication consumption in IBR patients. However, there is not enough evidence to draw strong conclusions regarding PVB’s impact on opioid consumption and LOS. Two of 3 studies reporting pain show PVB reduces acute postoperative pain (during the recovery room and within the first postoperative day following PMR) compared to GA alone.^36,37^ Furthermore, 4 of 5 studies indicate significant decreases in postoperative nausea and antiemetic consumption. There is positive evidence of PVB’s impact on opioid consumption. Lastly, there is not enough evidence to state PVB significantly reduces LOS with 4 of 6 studies showing no significant difference between PVB and non-PVB groups.^36,37,40,41^

The 2 RCTs show an overall significant decrease in acute postoperative opioid consumption,^37,38^ a finding supported by 2 retrospective studies also indicating significant decreases in opioid consumption among the PVB group.^38,41^ However, 1 study showed a decrease in opioid consumption but lacked statistical analysis,^39^ while another study conversely reports significantly higher consumption of opioids in the PVB group versus the control group receiving LB in the PACU.^40^ Abdelsattar et al did not use contemporary groups for comparison and found no difference in opioid consumption at later postoperative time points. Due to the strength of evidence from the included RCTs in addition to some evidence from observational studies, PVB does seem to reduce acute postoperative opioid consumption.

Several studies have shown that IBR patients are at risk for significant, postoperative pain compared to ABR patients.^6,8,50^ As such, these patients require more narcotics and other analgesics for their pain management.^7,8^ Suboptimal pain management is associated with further negative effects: slowed recovery, increased morbidity, prolonged opioid use during and after hospitalization, impaired physical function, and lowered quality of life.^8,51,52^ Increased, acute postoperative pain is a significant predictor of chronic pain syndromes, which negatively impact quality of life and affects nearly 50% of patients with breast cancer.^9–12,53,54^ One study reviewed long-term chronic pain, reporting PVB patients had less pain compared to GA alone at 3 months following PMR^37^; however, the mechanism for developing chronic pain among breast cancer patients following PMR is poorly understood.

Possible factors contributing to the development of chronic pain in this patient population may include: pain due to surgical scars, chest wall pain, upper-arm pain, shoulder discomfort, and/or a neuropathic component that develops into a complex, regional pain syndrome.^55^ Surgical technique is another possible mechanism for the development of chronic pain in IBR patients. The predominant technique, placement of the implant under the pectoralis muscle, has been associated with increased pain compared to the prepectoral method.^6,7^ Manipulation of the muscle could lead to higher rates of referred pain^7^ or muscle spasming.^55–57^ Lastly, psychological factors and psychological vulnerability to pain perception are significantly associated with greater postoperative pain intensity.^58,59^

The results of this systematic review support PVB’s value as an alternative to opioids for controlling postoperative pain among IBR patients. However, this review was limited by the quantity and quality of the available literature. The significant heterogeneity in the results prevented the conduct of a meta-analysis. Aufforth et al. found no significant difference in average pain scores on all postoperative days between the PVB and non-PVB groups, whereas Fahy et al. reported no significant difference in average pain scores between their experimental groups, on the day of surgery. However, Aufforth et al. did not specifically distinguish patients who only underwent IBR.^38^ As well, Fahy et al. did not distinguish between mastectomy only patients versus mastectomy plus reconstruction patients; nor did this study detail the scoring system used.^41^ For LOS, Aufforth et al. displayed a significantly lower LOS for the PVB group versus the non-PVB group and no significant difference in the proportion of patients with PONV. However, these results refer to all included patients, not simply IBR patients.^38^

The majority of the included studies were retrospective and observational. Other limitations of reviewed studies include: studies with small sample sizes, use of varying anesthetic agents for PVB injection along with different anesthetics for the control group, the lack of standard postoperative protocols for pain management, lack of controlling for the impact of sentinel lymph node biopsy or axillary lymph node dissection on pain, and different measurement scales for each outcome of interest. An additional limitation is lack of reported costs incurred from using PVB. From a policy perspective, understanding the costs of introducing an intervention is an important factor in considering its overall benefits and should be included in future studies.

In multiple meta-analyses that included patients undergoing an array of breast surgery procedures (from breast-conserving surgery to cosmetic), PVB has been shown to be a superior and effective treatment for postoperative pain management, limiting opioid consumption, and reducing LOS and PONV compared to GA alone.^31,32,60,61^ However, to draw strong conclusions about PVB’s effectiveness in IBR patients is dependent on further research. A systematic review conducted by Offodile et al. assessing the effectiveness of PVB in PMR (both IBR and ABR) patients was overall consistent with our findings. The review of Offodile et al. was limited by number and quality of included studies (9 studies were included, of which 7 were observational while 2 were RCTs) and heterogeneity of results. Therefore, they reported on the trends of PVB’s efficacy showing some positive impact in improving pain management, decreasing opioid consumption, and reducing LOS while not impacting PONV.^62^

Despite the limitations, this review is a rigorous synthesis of current literature. Overall, the limited number of studies and heterogeneity of results necessitates further research in examining PVB as an alternative to narcotic, postoperative pain control in the prosthetic PMR patient.

## CONCLUSIONS

Given the current opioid crisis, there is a growing emphasis on non-narcotic alternatives in the perioperative setting. In prosthetic PMR, PVBs may provide an alternative analgesic approach to improve postoperative pain and enhance recovery. Evidence suggests that PVB reduces acute, postoperative pain, improves PONV, and may have a positive impact on reducing opioid consumption. There is not enough evidence to support that PVB is associated with a decreased LOS. More high-quality studies are needed to assess the effects of PVB on perioperative opioid consumption, quality of recovery, and chronic pain in IBR patients.
